# Neuromuscular Blockade Antagonism for Thyroid Surgery During Intraoperative Neural Monitoring—An Anesthesia Perspective

**DOI:** 10.3390/medicina61030420

**Published:** 2025-02-27

**Authors:** I-Cheng Lu, Sheng-Hua Wu, Pi-Ying Chang, Tzu-Yen Huang, Che-Wei Wu, Po-Yang Chen

**Affiliations:** 1 Department of Anesthesiology, Kaohsiung Medical University Hospital, Kaohsiung Medical University, Kaohsiung 807, Taiwan; 2 Faculty of Medicine, College of Medicine, Kaohsiung Medical University, Kaohsiung 807378, Taiwancwwu@kmu.edu.tw (C.-W.W.); 3 Department of Anesthesiology, Kaohsiung Municipal Siaogang Hospital, Kaohsiung Medical University, Kaohsiung 812, Taiwan; 4 Department of Otolaryngology—Head and Neck Surgery, Kaohsiung Medical University Hospital, Kaohsiung Medical University, Kaohsiung 807, Taiwan

**Keywords:** thyroid surgery, intraoperative neural monitoring (IONM), neuromuscular blockade (NMB), sugammadex, neostigmine, anesthetic management

## Abstract

*Background and Objectives:* Thyroid surgery with intraoperative neural monitoring (IONM) of the recurrent laryngeal nerve (RLN) requires precise anesthetic management. This narrative review compares non-selective (neostigmine) and selective (sugammadex) reversal agents for neuromuscular blockade (NMB), discussing their mechanisms of action and the challenges of achieving optimal NMB reversal without compromising surgical conditions or IONM quality. *Materials and Methods:* A literature search was conducted using PubMed, MEDLINE, and Google Scholar for studies published up to November 2023. Relevant case studies, clinical trials, systematic reviews, and guidelines focusing on NMB reversal in thyroid surgery with IONM were included, prioritizing investigations involving sugammadex and neostigmine. *Results*: Clinical evidence indicates that sugammadex (0.5–1 mg/kg) provides the rapid and reliable return of neuromuscular function, benefiting electromyography (EMG) signal quality preservation. However, overshooting the reversal can precipitate patient movement, compromising surgical precision. Neostigmine (0.03–0.04 mg/kg), while less selective, remains a cost-effective alternative, with recent studies suggesting adequate support for IONM signal integrity when carefully dosed and timed. *Conclusions*: This review underscores the need for balanced NMB reversal strategies tailored to intraoperative monitoring requirements in thyroidectomy. Further randomized trials and large-scale studies are needed to refine and standardize NMB reversal strategies in thyroid surgery with IONM.

## 1. Introduction

Thyroid surgery is one of the most common endocrine procedures worldwide, with total or subtotal thyroidectomy performed for various thyroid pathologies, including malignancies and hyperthyroidism [1,2,3]. The preservation of the recurrent laryngeal nerve (RLN) function is essential to prevent postoperative vocal cord paralysis, dysphonia, and a compromised airway [4,5]. Intraoperative neural monitoring (IONM) has gained popularity as an important adjunct for protecting the RLN during thyroidectomy by providing real-time electromyography (EMG) signals [6,7].

Achieving high-quality EMG signals depends on the precise management of neuromuscular blockade (NMB) and its reversal. Neuromuscular blocking agents (NMBAs), also known as skeletal muscle relaxants, facilitate optimal intubation conditions and adequate surgical relaxation. However, excessive neuromuscular blockades can attenuate EMG signals, and insufficient neuromuscular blockades may lead to unwanted patient movement and coughing. The timing and dosing of neuromuscular blockade reversal must, therefore, be carefully managed to balance adequate surgical conditions with reliable neural monitoring [8].

This narrative review aims to provide a comprehensive analysis of the current literature on neuromuscular blockade reversal in thyroid surgery using IONM. We compare the pharmacologic mechanisms, clinical efficacy, and safety profiles of selective and non-selective reversal agents, with emphasis on emerging evidence regarding sugammadex and neostigmine. Finally, we discuss challenges, offer perspectives on Enhanced Recovery After Surgery (ERAS) protocols, and propose future directions for optimizing anesthetic and surgical outcomes.

## 2. Overview of Neuromuscular Blockade in Thyroid Surgery

### 2.1. Importance of Neuromuscular Blockade

Neuromuscular blocking agents (NMBAs) play a crucial role in thyroid surgery anesthesia, facilitating optimal intubation conditions, reducing laryngeal morbidity, and providing adequate surgical relaxation [9,10]. However, in thyroidectomies employing intraoperative neuromonitoring (IONM), the degree of neuromuscular blockade (NMB) must be precisely titrated to avoid interference with electromyography (EMG) signals from the recurrent laryngeal nerve (RLN) [11].

### 2.2. Impact on Intraoperative Neural Monitoring

IONM utilizes endotracheal tubes with surface electrodes to capture EMG signals from the vocalis muscle. Excessive neuromuscular blockade can suppress EMG signal amplitude, potentially leading to false-negative results for RLN injury. Conversely, insufficient neuromuscular blockade or over-reversal may result in patient movement, coughing, or bucking, compromising surgical visualization and instrument handling [12].

### 2.3. Rationale for Neuromuscular Blockade Reversal

The timely reversal of neuromuscular blockade is of key importance for several reasons. First, it preserves the EMG signal amplitude by ensuring adequate nerve–muscle transmission and reliable intraoperative signals. Second, it facilitates safe extubation by preventing a residual neuromuscular blockade that could otherwise lead to respiratory complications or delayed postoperative recovery [13]. Finally, proper reversal improves patient outcomes by reducing the risk of RLN damage and optimizing surgical workflows.

The management of neuromuscular blockade in thyroid surgery with IONM requires a delicate balance between providing optimal surgical conditions and maintaining the integrity of neural monitoring signals. This balance is achieved through the careful titration of NMBAs and their reversal agents, guided by quantitative neuromuscular monitoring.

## 3. Neuromuscular Blocking Agents and Antagonism for General Population

### 3.1. Neuromuscular Blocking Agents (NMBAs)

Non-depolarizing NMBAs act as competitive antagonists at the acetylcholine receptor, preventing acetylcholine from binding and thereby inhibiting neuromuscular transmission. These agents can be broadly classified into two chemical classes: aminosteroids (e.g., rocuronium, vecuronium) and benzylisoquinolines (e.g., cisatracurium and atracurium). In current practice, common intermediate-acting NMBAs for surgery include rocuronium and cisatracurium [14]. Rocuronium, a steroidal NMBA, has a relatively rapid onset but must be used cautiously because repeated dosing may result in prolonged blockade [15]. Atracurium and cisatracurium, which are benzylisoquinolinium compounds, undergo Hofmann elimination and ester hydrolysis, thus reducing their reliance on renal or hepatic clearance [16].

### 3.2. Neuromuscular Blockade Antagonism (Reversal Agents)

#### 3.2.1. Selective Relaxant Binding Agent—Sugammadex

Sugammadex is a modified γ-cyclodextrin designed to encapsulate aminosteroidal NMBAs (e.g., rocuronium, vecuronium), forming an inactive complex [17]. This mechanism is highly selective and does not rely on acetylcholine levels, thereby minimizing cholinergic side effects. Clinical trials have shown that sugammadex can rapidly reverse moderate or even deep rocuronium-induced neuromuscular blockade [18]. In a multicenter study of 89,753 surgeries, both sugammadex and neostigmine were associated with adverse events—including bradycardia, anaphylaxis, bronchospasm, and cardiac arrest—with sugammadex showing a slightly higher overall incidence (3.4% vs. 3.0%) and a marginally increased rate of bradycardia (2.4% vs. 2.2%) compared to neostigmine. These findings indicate that the two agents have comparable safety profiles [19].

Despite its higher acquisition cost relative to neostigmine, sugammadex was associated with USD 232 lower total expenditures in the matched cohort [20]. This cost benefit is likely attributable to sugammadex’s rapid reversal of the neuromuscular blockade, which shortens both surgical and recovery room durations. Such time-saving methods enhance operating room efficiency and reduce delays in patient transfer. Moreover, a faster recovery minimizes the risk of postoperative complications, including respiratory issues and residual blockade. Ultimately, fewer complications and shorter recovery periods contribute to lower direct hospital costs and improved patient throughput, especially in low-risk surgical populations.

#### 3.2.2. Non-Selective Relaxant Binding Agent—Cholinesterase Inhibitors

Neostigmine, a cholinesterase inhibitor, is a non-selective reversal agent with a broad-spectrum effect compared to sugammadex. Increasing acetylcholine concentrations at the neuromuscular junction exerts a nicotinic effect that counteracts the competitive blockade induced by both aminosteroids (e.g., rocuronium) and benzylisoquinolines (e.g., cisatracurium) NMBAs [21].

To mitigate muscarinic side effects such as bradycardia, it is commonly co-administered with anticholinergic agents (e.g., glycopyrrolate and atropine). Although it is widely accessible and cost-effective, its efficacy and speed of reversal can be unpredictable in cases of profound neuromuscular blockade. Moreover, excessive neostigmine can cause excessive parasympathetic stimulation if not adequately counteracted by anticholinergic agents [22]. Table 1 presents a comparison of the key characteristics between sugammadex and neostigmine [23,24,25,26].

## 4. Neuromuscular Blockade Antagonism During Intraoperative Neural Monitoring (IONM)

### 4.1. Rationale: Balancing IONM Quality and Surgical Conditions

A key challenge in thyroid surgery with IONM is achieving an optimal depth of neuromuscular blockade that preserves adequate EMG signal quality while maintaining favorable surgical conditions [27]. Both the under-reversal and over-reversal of neuromuscular blockade carry considerable risks:

- Under-reversal: This may result in diminished EMG signals, delayed or absent nerve identification, and elevated risks of recurrent laryngeal nerve (RLN) injury.
- Over-reversal: This may lead to patient movement, bucking, or coughing, potentially dislodging endotracheal tube electrodes and disrupting the surgical field [28].

### 4.2. Prior to Sugammadex

Before sugammadex became available, neuromuscular blockade management depended on the careful titration of rocuronium or atracurium doses or spontaneous recovery to enable reliable nerve signal detection [29]. Although feasible, this approach could prolong surgical steps if target nerve EMG signals remain suppressed. Alternatively, some anesthesiologists administer lower rocuronium doses (e.g., 0.3 mg/kg) to preserve EMG signals at the expense of suboptimal intubation conditions [30].

### 4.3. Following Sugammadex Implementation

Sugammadex has revolutionized neuromuscular blockade management by allowing the rapid reversal of aminosteroidal NMBAs at virtually any depth of blockade [17]. Early clinical reports indicated that a sugammadex dose of 2 mg/kg could effectively restore EMG signals but was associated with patient bucking rates ranging from 20% to 35% [31,32]. One ongoing challenge is to maintain high-amplitude EMG signals while preventing excessive patient movement. Recent studies suggest that lower doses (0.5–1 mg/kg) can achieve sufficient EMG signal recovery with reduced incidence of bucking [8,31]. A titrated approach to sugammadex, based on the actual depth of the neuromuscular blockade, may help optimize both patient outcomes and resource utilization [27].

Although many sugammadex reversal protocols are standardized (e.g., based on anesthesia induction or the timing of skin incisions), individual variations in surgical duration or NMB recovery may lead to suboptimal outcomes when fixed doses are used at preset time points. A “surgeon-centered” sugammadex protocol—delivering titrated sugammadex doses based on neuromuscular blockade monitoring at the moment of EMG testing—has been linked to high-quality IONM signals, predictable surgical relaxation, and enhanced safety [33]. This teamwork underscores the importance of flexible, tailored NMB management to improve both surgical efficiency and monitoring quality.

### 4.4. Neostigmine Repurposing

Neostigmine effectively antagonizes shallow or minimal neuromuscular blockade, often defined as a train-of-four (TOF) ratio ≥ 0.4 with a neuromuscular transmission monitor, and is traditionally used to reverse the residual neuromuscular blockade at the conclusion of surgery [21]. A recent porcine study found that neostigmine significantly shortened laryngeal EMG recovery time after a cisatracurium- or rocuronium-induced blockade [34]. Moreover, clinical trials combining neostigmine (2 mg) with glycopyrrolate (0.4 mg) yielded adequate EMG signals with a low incidence of bucking (4%, 2/50 cases), which was notably lower than the 13.7% (7/51) bucking rate reported with low-dose sugammadex [24,35]. These findings suggest that neostigmine-based protocols can align well with the timelines required for most thyroid surgeries, facilitating reliable EMG signals and minimizing unwanted patient movement (Table 2).

Although sugammadex offers distinct advantages, neostigmine remains a viable alternative in IONM settings where sugammadex may be limited by cost or regulatory constraints [36]. Further research is warranted to optimize the timing, dosing, and overall risk–benefit profile of using neostigmine in thyroid surgery with IONM.

## 5. Neuromuscular Blockade Management in Enhanced Recovery After Surgery (ERAS) Protocols

Enhanced Recovery After Surgery (ERAS) protocols aim to optimize perioperative care, reduce complications, and promote faster recovery [37]. Within the context of thyroid surgery, the principal components of ERAS protocols include the following: (1) preoperative optimization to improve patients’ physical and psychological readiness for surgery; (2) stress reduction strategies to mitigate surgical stress during the perioperative period; and (3) accelerated recovery approaches to facilitate early discharge, reduce medication use, and promote functional recovery [38,39].

The effective reversal of neuromuscular blockades plays a critical role in ensuring successful extubation and reducing the incidence of postoperative pulmonary complications [40,41]. Proper management minimizes the risk of a postoperative residual neuromuscular blockade, thereby enabling patients to regain adequate muscle strength to protect their airway post-extubation and resume normal activities. The successful implementation of an ERAS protocol requires close interdisciplinary collaboration among anesthesiologists, surgeons, and healthcare professionals. Future advancements may focus on the development of standardized guidelines for neuromuscular blockade reversal, specifically tailored to intraoperative neuromonitoring in thyroid surgery.

## 6. Conclusions

Optimal neuromuscular blockade and its reversal are crucial for effective IONM during thyroid surgery. While a deep neuromuscular blockade may compromise EMG signal fidelity, excessive reversal can undermine surgical conditions and patient safety. Sugammadex allows for rapid and precise reversal but requires judicious dosing and timing to prevent unintended movement. Neostigmine serves as a broad-spectrum reversal agent that remains a viable alternative because of its wide accessibility and cost-effectiveness, coupled with minimal patient movement and reliable EMG signals. Based on current clinical evidence, an individualized titrated sugammadex dose between 0.5 and 1 mg/kg effectively facilitated the rapid recovery of EMG signals while reducing patient movement. In parallel, administering neostigmine at a dose of 0.03–0.04 mg/kg, along with proper anticholinergic support, helped achieve an optimal balance between efficacy and safety.

One limitation of this study is its narrow focus on thyroid surgery with IONM, which may not capture the full spectrum of current practices. This study also did not include a survey or poll including academic medical centers, thereby limiting our insight into real-world usage patterns and innovative practices beyond the literature. Future research incorporating multi-institutional surveys and expanding the investigation to other surgical fields could provide a more comprehensive and broadly applicable perspective.

Further large-scale studies are warranted to establish standardized NMB reversal guidelines, with the ultimate goal of enhancing patient safety, preserving RLN integrity, and improving postoperative outcomes in monitored thyroidectomy.

Despite significant strides in neuromuscular blockade reversal, the following issues deserve future investigation: (1) Optimal reversal dosing: further randomized controlled trials are needed to compare various sugammadex and neostigmine dosing strategies tailored to neuromuscular monitoring results. (2) Safety in specific populations: individuals with morbid obesity, chronic obstructive pulmonary disease, or neuromuscular disorders may have different reversal requirements. (3) Cost-effectiveness: while sugammadex is highly effective, its cost and availability limit its universal adoption. Studies that evaluate cost–benefit ratios in different healthcare settings can guide rational use [36]. (4) Long-term outcomes: research correlating immediate postoperative IONM results with long-term voice and swallowing outcomes may further validate the utility of NMB reversal regimens [42].

## Figures and Tables

**Table 1:** Comparison of the key characteristics between sugammadex and neostigmine.

	Sugammadex	Neostigmine
Mechanism of Action [23]	Encapsulates and inactivates rocuronium and vecuronium by forming tight complexes, leading to the rapid reversal of neuromuscular blockade.	Inhibits acetylcholinesterase, thereby increasing acetylcholine levels at the neuromuscular junction, which competitively reverses blockade.
Speed of Reversal for Extubation [23]	Provides rapid reversal even for deep neuromuscular blockade.	Reversal is generally slower and is only effective in minimal neuromuscular blockade.
Impact on Intraoperative EMG Signals [24,25]	Minimal interference with EMG signals, ensuring more stable and reliable neuromonitoring during surgery.	May cause fluctuations in EMG signals due to increased acetylcholine levels; the EMG signals were effectively obtained in a pilot study.
Cost	Typically has a higher cost compared to neostigmine.	Generally, it is a more cost-effective option.
Adverse Effects [23,26]	Generally, it is well tolerated; potential adverse effects include rare cases of hypersensitivity reactions and bradycardia.	May cause common side effects such as bradycardia, nausea, increased salivation, and other cholinergic effects.

EMG = electromyography.

**Table 2:** A study investigating the effect of neostigmine on laryngeal electromyography during thyroid surgery.

Authors and Year	Study Type	Group and Size	NMBA Dose	Reversal Dose	Reversal Timing	Key Findings
Oh et al., 2021 [24]	Retrospective	*n* = 50	Rocuronium 0.6 mg/kg	Neostigmine 2 mg Glycopyrrolate 0.4 mg	Nearly 5 min after NMBA	Mean V1 amplitude: 985.3 ± 471.6 μV. All patients V1 > 500 μV. Mean time to twitch: 21.0 ± 4.5 min
Lu et al., 2022 [34]	Prospective (Porcine)	Rocuronium *n* = 4 Cisatracurium *n* = 4	Rocuronium (0.6 mg/kg) Cisatracurium (0.2 mg/kg)	Neostigmine 0.04 mg/kg	10 min after NMBA	Recovery time to 50% EMG: 16.5 min. Maximum recovery: ~85%
Oh et al., 2022 [35]	RCT	Neostigmine (*n* = 20) Saline (*n* = 20)	Rocuronium 0.6 mg/kg	Neostigmine 0.03 mg/kg Glycopyrrolate 0.006 mg/kg	Nearly 5 min after NMBA	The neostigmine group had a faster V1 signal (15.4 vs. 19.9 min) and higher V1 first-check success (100% vs. 50%)

RCT = randomized control trial; NMBA = neuromuscular blocking agents; V1 = initial vagal stimulation to obtain electromyography signal during thyroid surgery; EMG = electromyography

## Data Availability

No new data were created or analyzed in this study.
